# Exploiting Chemistry to Improve Performance of Screen-Printed, Bismuth Film Electrodes (SP-BiFE)

**DOI:** 10.3390/bios6030038

**Published:** 2016-07-22

**Authors:** Carlo Dossi, Damiano Monticelli, Andrea Pozzi, Sandro Recchia

**Affiliations:** 1 Dipartimento di Scienze Teoriche e Applicate (DiSTA), University of Insubria, Via Dunant, 2, 21100 Varese, Italy; 2 Dipartimento di Scienza ed Alta Tecnologia (DiSAT), University of Insubria, Via Valleggio, 11, 22100 Como, Italy; damiano.monticelli@uninsubria.it (D.M.); andrea.pozzi@uninsubria.it (A.P.); sandro.recchia@uninsubria.it (S.R.)

**Keywords:** bismuth film electrodes, screen-printed electrodes, bismuth chemistry, metal/surface interactions, surface modifications

## Abstract

Mercury substitution is a big issue in electroanalysis, and the search for a suitable, and less toxic, replacement is still under development. Of all the proposed alternatives, bismuth films appear to be the most viable solution, although they are still suffering some drawbacks, particularly the influence of deposition conditions and linearity at low concentrations. In this paper, the most promising strategies for bismuth film deposition on screen-printed electrodes (surface modifications, polymeric film deposition, insoluble salt precursors) will be evaluated for trace metal analysis. Particular attention will be devoted to bismuth chemistry, aiming to rationalize their electroanalytic performance.

## 1. Introduction

Anodic stripping voltammetry (ASV) has always been regarded as an extremely sensitive electroanalytical technique, exploiting the properties of certain electrodic materials to preconcentrate analytes, both inorganic and electroactive organic molecules, on their surface and analyze them by stripping back to the bulk of solution. Since the beginning, mercury played a central role, thanks to the ease of preparation and its good analytical figures in electroanalysis, particularly reproducibility and sensitivity with heavy metal determination [1,2,3]. However, the well-known toxicity of mercury and of its salts used for the preparation of the film electrode is increasingly posing environmental and safety concerns on their use. Therefore, researchers have been trying for a long time to find new materials and new preparation strategies to the development of reliable and environmentally-friendly non-mercury electrodes for on-site monitoring of heavy metals and organic molecules, in both pollution and pharmaceutical fields.

Materials such as gold [4], iridium [5], antimony [6], or boron-doped diamonds [7,8] have been investigated. In general, their performances have not approached those of mercury, either in terms of reproducibility and overall analytical performances.

On the contrary, since the pioneering works by Wang and Hocevar [9,10], bismuth has drawn considerable interest as a soft metal to prepare electroactive surface films, because of its remarkable low toxicity and its ability to form alloys with many metals, as well as its partial insensitivity to dissolved oxygen, and a quite wide potential window which is necessary for stripping techniques. 10 years after the discovery of bismuth film electrodes, a review paper [11] reported 222 references of research papers devoted to bismuth film preparation and application in electroanalysis. Studies have been chiefly devoted to trace metal analysis, given the scientific and practical interest in developing simple, environmentally-friendly methods for in situ monitoring of these pollutants. Recently, carbon nanotubes and graphene nanostructures are increasingly being used to improve their performance and ease of use in the field, as well as the use of macroporous structures [12,13,14]. Research on electroanalytical studies of organic molecules have been carried out as well, as it is demonstrated in a recent review [15].

A survey on all this body of literature evidenced that a broad range of different techniques have been proposed for the preparation of the active bismuth film, and particularly on screen printed electrodes, which seems to be particularly sensitive to the nature of the bismuth precursor, the occurrence of kinetics limitations, and the sensitivity to the carbon surface cleanliness [16,17,18]. Moreover, surface modification with polymers, Nafion being the most commonly used, is commonly exploited to alleviate interferences and improve the mechanical stability of the bismuth film [19,20]. All these observations might be explained on the basis of the rich and complex chemistry of bismuth in aqueous solution [21]. On one side, bismuth(III) ions easily coordinate with oxygen-based ligands, such as polycarboxylic and polyaminocarboxylic acids, leading to the formation of polymeric and monomeric bismuth(III) complexes [22]. The ion-exchange groups of the protecting polymer might, therefore, play a chemical role during the preparation of the bismuth phase. Moreover, the formation of insoluble basic BiOX (X = Cl^−^, Br^−^, NO_3_^−^ etc.) species may easily occur in aqueous solution of the bismuth salts commonly used for the electrochemical deposition, and great care must be exercised, since precipitation as a colloidal form is often observed.

In this paper, surface modifications of the carbon working electrode, the nature of the polymeric film cast onto the working electrode, and the formation of insoluble salt precursors have been investigated and rationalized as the most promising strategies for the preparation of screen-printed bismuth film electrodes (SP-BiFE). These electrodes were then tested in the stripping analysis of cadmium and lead as model analytes to test surface-bismuth interactions, and to evaluate their analytical performance in trace metal analysis. The understanding of bismuth chemistry was able to give important information for rationalizing the behavior of the bismuth film as a function of the preparation strategy, both for trace inorganic analysis and for bioanalytics.

## 2. Materials and Methods

### 2.1. Apparatus and Reagents

Screen-printed electrodes (SPEs) have been a kind gift by Prof. Giuseppe Palleschi and his group at the University of Roma “Tor Vergata”. Electrodes have been printed on a flexible polyester film. A graphite-based ink (Elettrodag 421) from Acheson (Milan, Italy) was used to print the working and counter electrode, and a silver ink was used to print the pseudo-reference electrode. The diameter of the working electrode was 0.3 cm, resulting in a working area of 0.07 cm^2^. The printing procedures have been already described in the literature [23,24,25].

For electroanalytical experiments, a portable PalmSens instrument (Palm Instruments BV, the Netherlands) and an Amel 4330 versatile electroanalytical instrument (Amel srl, Milano, Italy) were used. The Amel 4330 instrument proved very effective for stripping analysis, because of its integrated voltammetric cell, which enables, when required, automatic stirring and degassing throughout all the electroanalytic steps. A specially designed cell was devised to optimize mass transport to the electrode and prevent water or acid vapors to degrade the electric contacts. This cell allowed the use of an external 3 M KCl Ag/AgCl reference electrode in order to reference all potentials. In those cases where the internal pseudo-reference Ag wire was used, a 0.01 M chloride concentration in the cell solution was used, and potentials were referred to the reference electrode.

The two instruments were interfaced to a Windows-based personal computer via VApeak software. All instrument control, as well as full data analysis (peak finding, peak height and area calculations, calibration, and standard addition curves) are handled by the VApeak software, requiring no external modules.

All quantitative calculations are done on peak areas measured as (μA * mV). Quantitative data are interpolated by best fit calibration line which is reported as *y* = m*x* + q, where *y* is peak area and *x* is the concentration in μg/L. Analytical sensitivity was defined as the slope (**m**) of the best fit calibration line, reported as (μA * mV)/(μg/L). The intercept (**q**) is reported as peak area. Limits of detection (LOD) were determined when necessary, and calculated as 3σ/m, where σ was determined by running 10 replicate analyses on a standard sample near the limit of detection, and m is the analytical sensitivity. Limits of detection are reported as μg/L.

All the PTFE beakers and cells, round-bottomed flasks in polypropylene, polypropylene tubes, and pipette tips used for preparation and storage of samples and solutions were rinsed with copious amounts of double-distilled water. Solutions were prepared with double-distilled water (Milli-Q system, Millipore).

Hydrochloric and nitric acid (TraceSelect from Fluka) were purified by sub-boiling distillation in a quartz-Teflon apparatus. Sodium carbonate and sodium hydroxide were obtained from Fluka. Sodium acetate and sodium chloride (Trace Select) were purchased from Fluka. Disodium hydrogen phosphate and sodium metavanadate were purchased from Aldrich.

Cadmium and lead standard stock solutions (1000 mg/L) were from Sigma–Aldrich and diluted as required. Bismuth stock solution (1000 mg/L) was prepared from bismuth nitrate pentahydrate (CarloErba) in nitric acid.

Nafion perfluorinated ion-exchange resin, 5 wt % solution in lower aliphatic alcohols/water was purchased from Aldrich. Methocel 90HG (22%–27% methoxyl bases, Fluka) and Poly(sodium-4-styrene sulfonate) (PSS, Aldrich) were used as such.

High-purity MilliQ water (from MilliPore, Darmstadt, Germany) was used for all solutions and standards.

### 2.2. Measurement Procedures

Prior to the analysis, screen-printed electrodes were cleaned in ethanol, rinsed in water, and left in a 0.01 M hydrochloric acid solution. No problems of lead or cadmium contamination were found.

Electrodes were used in a standard three-wire configuration using the screen-printed working and counter carbon electrodes, and an external Ag/AgCl (3 M KCl) electrode as reference. The internal silver wire was not used as pseudo-reference electrode, since the chloride ion activity could not be kept constant throughout all experiments.

### 2.3. Oxidative Pretreatment of the Screen-Printed Electrodes

Three types of screen-printed electrodes were prepared and tested:
**No treatment**.SPEs were used as received, after standard cleaning. No unwanted lead signals were observed in the stripping analysis.**Treatment A (Type A)**.The screen-printed electrode is preoxidized at +1.50 V in 0.1 M acetate buffer at pH = 4.4 for 120 s, following what proposed by Amine et al. [12].**Treatment B (Type B)**.The screen-printed electrode is preoxidized under basic conditions in a saturated solution of sodium carbonate at +1.20 V for 240 s.


After the oxidative treatment has been applied, the electrode is then dipped in an acetate buffer solution at pH = 4.4 with a bismuth concentration of 0.1 mM; the film is then obtained by reduction at −1.20 V for 30 s, under conditions where no hydrogen evolution was observed. Immediately after bismuth film deposition, the Nafion protecting layer is deposited, by drop-casting 1 μL of the Nafion solution onto the carbon surface of the working electrode. After drying in air, the electrode should be immediately used for the analysis. In fact, surface oxidation of the bismuth film is taking place during drying and Nafion casting, and the electrodes cannot be stored as such. Moreover, final results may be dependent on the experimental conditions; a careful standardization of experimental conditions is suggested to obtain good reproducibility.

The analysis of cadmium is performed in acetate buffer using differential pulse stripping. Deposition was done at −1.20 V for 60 s at a stirring rate of 300 rpm. Analysis was done between −1.05 V and −0.25 V with step potential of 5 mV, a pulse time of 100 ms, and pulse amplitude of 50 mV. Prior to analysis, the electrode is always cycled eight times in blank acetate buffer solution.

### 2.4. In Situ Preparation of Bismuth Film With Different Polymeric Layers

Untreated screen-printed electrodes, after standard cleaning, have been modified by drop-casting 5 μL of the bismuth/polymer solutions that have been prepared as follows:
**Bi/Naf**:a 5 mM solution of Bi(NO_3_)_3_ in nitric acid is mixed with the Nafion solution in a 1:10 ratio.**Bi/Meth**:100 mL of the 5 mM solution of Bi(NO_3_)_3_ in nitric acid are added with 50 mg of Methocel 90 and 80 mg of sodium acetate.**Bi/PSS**:100 mL of the 5 mM solution of Bi(NO_3_)_3_ in nitric acid are added with 516 mg of poly(sodium-4-styrene sulfonate) (PSS) to obtain a 25 mM solution of PSS as monomeric units.


After drying in air, the electrodes are dipped in blank solution of 0.01 M HCl (pH = 2) or 0.1 M acetate buffer (pH = 4.4). Deposition of the bismuth film was done by cycling eight times under the same experimental conditions used for analysis. Differential pulse stripping for the analysis of cadmium and lead was done with the same experimental conditions used before.

### 2.5. Preparation of Bismuth Films From Insoluble Salt Precursors

Untreated screen-printed electrodes, after standard cleaning, have been modified via deposition of the polymer layer and of an insoluble bismuth salt, either bismuth phosphate or bismuth vanadate.

The use of Nafion was only done with bismuth phosphate following the procedure reported in the literature [18], and named as (A):
**Bi/PO_4_/PSS(A)**Equal volumes of solutions of Na_2_HPO_4_ (4 mM), Bi(NO_3_)_3_ (1 mM), and Nafion are mixed. Then, 5 μL of this solution is drop-casted onto the working electrode.


For PSS, two additional preparation methods, named (B) and (C) have been investigated in order to have a better insight into the chemistry of bismuth/PSS interactions. Moreover, two different bismuth loadings were also prepared when necessary:
**Bi/PO_4_/PSS(A)**Equal volumes of solutions of Na_2_HPO_4_ (4 mM), Bi(NO_3_)_3_ (1 mM), and PSS (25 mM) are mixed. Then, 5 μL of this solution is drop-casted onto the working electrode.**Bi/VO_4_/PSS(A)**Same as Bi/PO_4_/PSS(A), except using 3 mM NaVO_2_ instead of 4 mM Na_2_HPO_4_.**Bi/PO_4_/PSS(B)**2 μL of the 25 mM solution of PSS is drop-casted onto the working electrode and left drying. Then, 5 μL of a solution prepared by mixing equal volumes of Na_2_HPO_4_ (4 mM) and Bi(NO_3_)_3_ (1 mM) is drop-casted onto the working electrode.**Bi/VO_4_/PSS(B)**Same as Bi/PO_4_/PSS(B), except using 3 mM NaVO_2_ instead of 4 mM Na_2_HPO_4_.**Bi/PO_4_/PSS(B5)**2 μL of the 25 mM solution of PSS is drop-casted onto the working electrode and left to dry. Then, 5 μL of a solution prepared by mixing equal volumes of Na_2_HPO_4_ (20 mM) and Bi(NO_3_)_3_ (5 mM) is drop-casted onto the working electrode.**Bi/VO_4_/PSS(B5)**Same as Bi/PO_4_/PSS(B5), except using 15 mM NaVO_2_ instead of 20 mM Na_2_HPO_4_.**Bi/PO_4_/PSS(C)**5 μL of a solution prepared by mixing equal volumes of Na_2_HPO_4_ (4 mM) and Bi(NO_3_)_3_ (1 mM) is drop-casted onto the working electrode and left to dry. Then, 2 μL of the 25 mM solution of PSS is drop-casted onto the working electrode**Bi/VO_4_/PSS(C)**Same as Bi/PO_4_/PSS(C), except using 3 mM NaVO_2_ instead of 4 mM Na_2_HPO_4_.


Thanks to the stability of the BiPO_4_ and BiVO_4_ phases, these solid-state based electrodes are indefinitely stable in air at room temperature after preparation. Prior of their use, the bismuth film is formed (“activation step”) by cycling the electrodes eight times in blank solutions of 0.01 M HCl (pH = 2) or 0.1 M acetate buffer (pH = 4.4) under the electroanalytical conditions for differential pulse stripping analysis (see below). After activation and formation of the active bismuth film, the electrodes cannot be stored in air.

The vanadium signal was monitored during the activation step, in order to understand the kinetics of BiVO_4_ particles decomposition. For BiPO_4_, a preliminary investigation using Laser Ablation, Inductively Plasma Mass Spectrometry (LA-ICP-MS) was started using a Nd:YAG laser at 266 nm (New Wave UP 266, Fremont, CA, USA) interfaced to ICP-MS (X Series II, Thermo Electron Corporation, Waltham, MA, USA).

The analysis of cadmium and lead is performed in acetate buffer at pH = 4.4 for the Nafion-based electrodes or in 0.01 M HCl for PSS-based electrodes, using differential pulse stripping. Deposition was done at −1.05 V for 60 s at a stirring rate of 300 rpm. Analysis was done between −0.95 V and −0.25 V with step potential of 5 mV, a pulse time of 100 ms, and pulse amplitude of 50 mV.

## 3. Results and Discussion

### 3.1. Chemical Pretreatments of the Carbon Surface

A chemical treatment of the carbon surface has been attempted, in order to improve sensitivity and linearity of bismuth film screen printed electrodes. This approach has been already reported by some authors, using electrochemical oxidation under acetate buffer conditions [12]. A novel treatment of carbon surface of SPE has been proposed under basic pH conditions, using saturated sodium carbonate solutions under oxidizing conditions at +1.2 V for 4 min. Generally, these oxidative pretreatments are claimed to remove any binder residues left on the carbon surface after the curing process. Actually, this is not of any use here, since the carbon surface of the screen-printed electrodes was already found to be very clean for our analytical purposes. Instead, the basic solution is reacting with the carbon surface, with the formation of –COO^−^ Na^+^ moieties. Such carboxylic surface groups will chemically graft the Bi^3+^ ions to the conductive carbon surface of the screen printed electrode. Bismuth ions are well known to have a strong oxophilic character, and this interaction should then result in an increased reducibility to bismuth metal.

**Figure 1:**
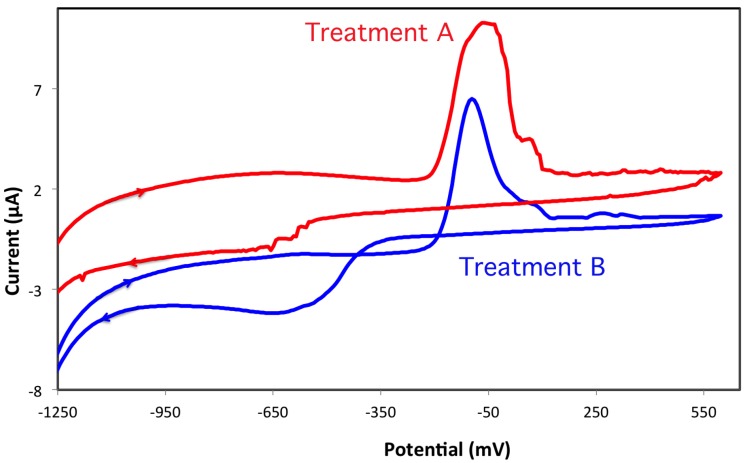
Cyclic voltammetry profiles of bismuth on pretreated screen-printed electrodes.

This suggestion has been investigated in cyclic voltammetry studies, where the two types of surface modification have been compared in Figure 1.

The cyclic voltammetric profiles are very similar for both electrodes, with an oxidation maximum around −60 to −100 mV, and a broad reduction peak is around −600 mV. This broad feature is indicative of kinetically limited reduction processes of Bi^3+^ to Bi^0^. However, the electrode treated under basic conditions shows a more definite electrochemical behavior, with narrower peaks, and a ratio between the oxidation/reduction peak areas, which is closer to unity. It may be concluded that the pretreatment under strong basic conditions tends to facilitate reduction of bismuth. This enhanced reducibility may be due to a better grafting of bismuth salt precursor to the graphite surface, leading to a more efficient electron transfer. However, the oxidative treatment, particularly when it is done under basic sodium carbonate solutions, resulted in a decreased mechanical stability of the graphite surface of the working electrode. Therefore, a Nafion coating was applied onto the treated surface after the bismuth film has been deposited. In this way, the electrode is mechanically stable and may withstand many analysis cycles in acidic conditions.

These electrodes have been then used for cadmium detection at ppb level under differential pulse stripping analysis. Prior the analysis, the electrodes are cycled in a blank solution under the experimental conditions of the stripping analysis, in order to re-reduce and stabilize the zerovalent bismuth film.

The analytical figures obtained from the calibration curves are reported in Table 1, where they are compared to a BiFE electrode, which has not been subjected to any type of surface pretreatment.

**Figure 2:**
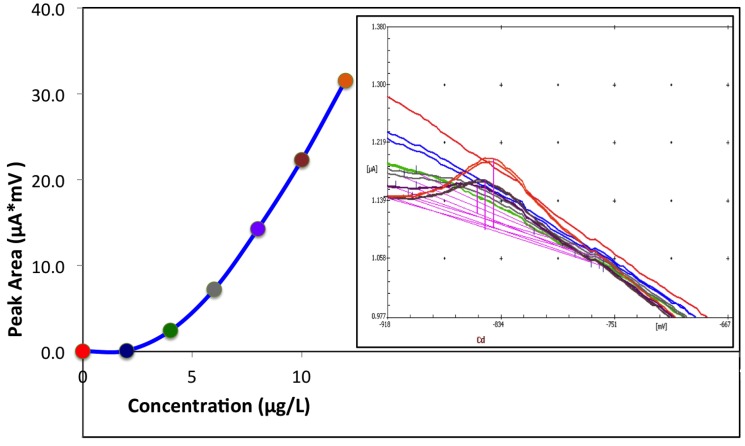
Non-linear calibration curve of cadmium analysis, and the corresponding ASV curves, at low concentrations (2 μg/L each addition) with a Type-B BiFE.

The first evidence from Table 1 is the effect of the chemical pretreatment of the graphite surface of the working electrode. A significant increase in the analytical sensitivity of more than three times for the treatment A and almost six times for treatment in sodium carbonate (Type B) is observed. This effect may be rationalized on the results found in cyclic voltammetry. The increased interaction between the bismuth film and the treated graphite surface leads to a more dispersed, more active bismuth surface, which is quite effective in reversibly optimizing the preconcentration and the following stripping of the analyte. However, the intercept of the best fit regression line is always negative, confirming that these bismuth films show evident non-linearity at low analyte concentrations. This behavior is well evidenced in Figure 2, where the differential pulse volktammograms and the calibration curve have been investigated at cadmium concentrations between 0 and 12 μg/L, using 6 additions of a 2 μg/L standard solution.

The experimental curves are quite noisy, and peak parameters are best calculated manually. Then, a non-linear curve is needed to interpolate the experimental data, since the linear portion of the curve starts to appear above 10 μg/L.

Interestingly, if the calibration curves of the three bismuth electrodes in Table 1 are calculated by discarding the first point, and only the calibration points above 12 μg/L are used, the slope is not significantly perturbed and a correct intercept value near zero is found. This non-linearity of bismuth alloys at low analyte concentrations might be tentatively ascribed to the restricted diffusion of the alloy bismuth phase with the metallic analyte, which should be less evident, or even disappear as in our case, at higher concentrations. The value found at the X-axis intercept decreases with the type of treatment, resulting in 4.6 μg/L for the untreated electrode, 1.87 μg/L for the type A treated electrode, down to 1.19 μg/L for the type-B treatment. It may be concluded that the chemical interaction between oxidative moieties and/or Na^+^ cations with the bismuth film is beneficial both to analytical sensitivity and to non-linearity at low concentrations.

However, these pretreatments, particularly the B-type, are sensitive to aging under atmospheric conditions. After a three-days’ exposure of the pretreated electrode to air, the analytical sensitivity dropped from 3.130 to 2.576 for the type-A electrode, and from 5.893 to 1.480 for the type-B electrode. This 74% loss of sensitivity for the electrode pretreated in sodium carbonate solution is detrimental for its practical application, and confirms the results by Amine et al. on the advantage of the electrode pretreatment under mild conditions at pH 4.3 [12] It may be inferred that bulk reoxidation of the bismuth film by atmospheric oxygen is favored for the more active, Type-B surface, which is likely to cause the growth of large Bi_2_O_3_ islands on the surface. Such large Bi_2_O_3_ structures cannot reform the nanosized, uniform bismuth film that was previously formed by electrochemical reduction of aqueous bismuth ions.

### 3.2. Investigation of the Chemical Effects of Polymer Coatings on Bismuth Films

These results prompted us to investigate whether such interactions may be shifted from the graphite surface to the active sites of polymeric film casted upon the formation of the bismuth film. Obviously, all these studies have then been performed on untreated screen-printed electrodes, without any prior surface treatment of the graphite surface of the working electrode. In this way, the coexistence of possible synergic effects between different oxygen moieties are avoided, as well as preferential electron transfer mechanisms to the modified carbon surface.

Three different polymers have been used, the commonly-used Nafion, Methocel [26], and the sodium salt of polystyrene sulfonate (PSS) [27], trying to modulate the chemical interactions between the bismuth salt precursor and the polymer. The Bi(NO_3_)_3_ precursor has been dissolved into the polymer solution prior its casting onto the graphite surface of the working electrode, in order to maximize their interaction. As it was observed with metal/oxide catalytic surfaces [28,29], oxophilic metal ions may tend to graft themselves to the oxygen ligands of the surface, with an optimal distribution on the surface, preventing the growth of large particles after casting. Then, the zerovalent bismuth film was obtained by performing eight blank cycles under the same experimental conditions used for the stripping analysis (see Experimental section).

The three different electrodes, named Bi/Naf, Bi/Meth, and Bi/PSS, have been tested for cadmium analysis at two different pH, at pH = 2 in 0.01 M HCl, and at pH = 4.4 in a 0.1 M acetate buffer. Interestingly, the electrodes behaved differently. Bi/PSS electrodes showed optimal conditions at pH = 2, whereas at pH = 4.4 the analytical signals were lower and not reproducible. Instead, the Bi/Naf and the Bi/Meth electrodes showed an inverse trend, with good and reproducible signals at pH = 4.4. However, a broad signal around −800 mV is observed in all experiments using Methocel as polymer, which prevents a good quantitation of the cadmium signal, which is observed in the same potential range. Since the presence of cadmium can be excluded in the pure Methocel polymer [26], a possible explanation has to be linked to the interaction of bismuth nitrate with the Methocel surface active sites, leading to an electroactive signal in the −800 mV region. Consequently, it has been decided to extend the analytical investigation also to lead, in order to gain more information on the electroanalytical behavior and the possibility of comparing data for all three types of bismuth-polymer surface.

The results of the calibration data on Cd and Pb analysis are then reported in Table 2, using pH = 4.4 for Nafion and Methocel and pH = 2 for PSS.

Interestingly, the slope of the calibration line for cadmium (1.098) is very similar to that found for the untreated electrode of Table 1 (1.008). This difference cannot be only ascribed to intrinsic variation of the chemical properties of the bismuth film. In fact, inter-electrode reproducibility and repeatability around 10% can be safely proposed, although they were often found to be better than this value by optimizing manual deposition of reagents by the operator. A significant improvement can, however, be obtained by the use of an automated liquid handling system.

The possible occurrence of cadmium/lead interactions in the stripping analysis of cadmium was reported on boron-doped diamond electrodes [7,8]. Here, any effect of lead to an increase of the cadmium signal is never observed in Nafion-coated bismuth electrodes; however, further studies will be required to fully support this hypothesis.

The effect of the chemical structure of the polymer surface is immediately evidenced in Table 2. First of all, Methocel appears to be unsuited to form and stabilize the bismuth film for trace metal analysis. Cadmium analysis cannot be safely run for the unwanted background signal, and the analytical sensitivity for lead is very low. Conversely, the sodium salt of polystyrene sulfonate seems very suited; comparing the analytical sensitivities with Nafion, a 2.5-fold increase of sensitivity for cadmium and a 4-fold increase for lead are observed. In the case of Bi/Naf electrode, analytical sensitivities for 1-min deposition seem comparable to those reported by Kefala et al. [19]; the intercepts were smaller, indicating a better linearity at low concentrations for Nafion-coated bismuth-film electrodes on glassy carbon. Limits of detections can be considered comparable, too, considering that a 10-min deposition step is used in Kefala’s paper.

More investigations on the actual chemistry taking place are necessary, but it may be proposed that the presence of RSO_3_^−^… Na^+^ or of RCOO^−^… Na^+^ moieties may optimize the kinetics of the bismuth film to reversibly form, during the preconcentration/stripping steps, a surface alloy with lead and cadmium, leading to optimal analytic behavior. However, a negative intercept is still observed for cadmium, which is particularly evident for the Bi/PSS electrode. This is a serious drawback for PSS-based electrodes, and the use of Nafion-based SPEs might be favored for trace analysis. However, the use of the polystyrene-sulfonate polymer should be further investigated, since its enhanced analytical response and the ability to work at low-pH conditions could open new analytical perspectives.

We conducted a search of the literature about the possible chemical alternatives in bismuth film deposition, with the aim to improve the non-linearity of the calibration curve in the low-concentration regime. The approach of Brainina et al. [18] to form the bismuth film using an insoluble bismuth salt (BiPO_4_) as bismuth precursor was found very promising here. Moreover, Nafion has been used to improve adhesion and mechanical stability of the film, as well as protector from surfactant fouling effects.

We therefore decided to reinvestigate such chemical approach to different polymer layers, since a chemical influence on the analytical performance of the bismuth electrode is to be expected, and the correct choice of the polymer may extend the usability of SP-BiFEs under acidic conditions.

The Nafion-BiPO_4_ layer has been prepared by depositing a drop of a mixture of Na_2_HPO_4_, bismuth nitrate and Nafion onto the graphite surface, allowing a slow drying to make a careful precipitation of the BiPO_4_ nanophase in Nafion. Based on the electrochemical redox behavior of the BiPO_4_ phase, which is in full agreement with that reported in [18], the bismuth active layer can be formed and activated by performing eight blank cycles at the desired pH. This pretreatment is different from that proposed by Brainina, and it was done to ensure a comparison with conventional electrodes prepared by electrochemical bismuth deposition. Our electrodes show good reproducibility in Cd and Pb analysis only working at pH = 4.4. The profiles recorded at pH = 2 were not reported, since a continuous increase of the stripping peaks is observed and no reliable data can be obtained.

**Figure 3:**
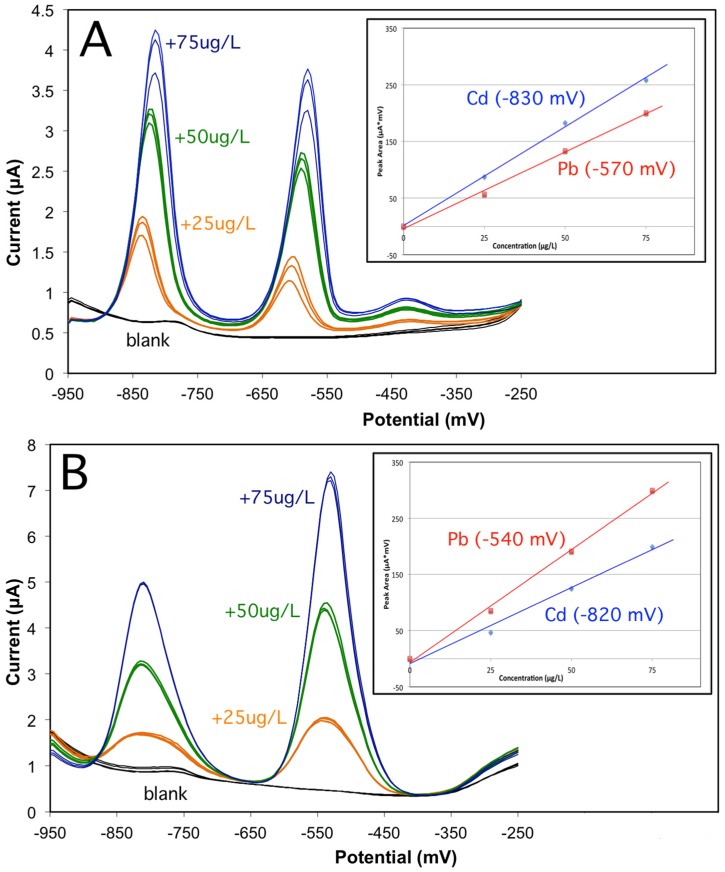
Differential-pulse stripping analysis of cadmium and lead with (**A**) Bi/PO_4_/Naf at pH = 4.4 and (**B**) Bi/PO_4_/PSS(A) at pH = 2. Calibration curves for Cd and Pb are shown in the insert.

A cleaning step at −200 mV at the end of the voltammetric analysis was then applied; no effect on this carryover effect was found at pH = 2, whereas at pH = 4.4, a small improvement on peak reproducibility was observed. This behavior is in very good agreement with that observed previously, and it prevented us from obtaining any reasonable quantitative data at pH = 2. At pH = 4.4, (Figure 3A), sharp and symmetric peaks were observed for cadmium around −830 mV and for lead around −570 mV. Moreover, the sensitivities for Cd and for Pb are in very good agreement with those obtained by electrochemical deposition of the bismuth film on the pre-activated surface using treatment A (see Table 1).

A Bi/PO_4_/PSS electrode was then prepared following the same experimental methodology that had used for the Bi/PO_4_/Naf electrode. The behavior of the PSS-based electrodes was, however, to be only studied at pH = 2, since the analytical response is very low at pH = 4.4, as it was previously observed. A typical analysis of Cd and Pb for three sequential additions of a 25 ug/L solution of Pb and Cd in a 0.01 M HCl is then reported in Figure 3B. The background signal is low and stable, and the reproducibility of the analytical signals is very good for all additions. Differently from the Bi/PO_4_/Naf electrode, no significant carryover effects were observed at this low pH; therefore, no cleaning step at the end of the analysis was required. The voltammetric peaks are broader and the separation between Cd and Pb is increased.

It might be proposed that, under low-pH conditions, restricted diffusivity of Pb and Cd ions is observed inside the polymer layer by electrostatic charging of the Nafion film, causing a carryover effect, with an irreproducible and continuous increase of Pb and Cd concentration at the graphite/polymer interface. On PSS, instead, the decreased diffusivity of metal ions is simply resulting in peak broadening. An inverse sensitivity between cadmium and lead is observed, but it is supposed to be related to the different operating pH.

A systematic investigation of the effect of the preparation method on the voltammetric properties of Bi-PSS electrode from BiPO_4_ precipitation has been undertaken, following the preparation methods named (A), (B), and (C) that are described in the Experimental section.

The three electrodes are then reduced by the standard activation method and tested for the analysis of cadmium and lead, using the Bi/PSS electrode as reference. The results are then reported in Table 3.

Interestingly, the sensitivity of both lead and cadmium analysis is dependent on the method used for the preparation of the active phase. It is immediately evident that the Bi/PSS electrode reported in Table 2, where no BiPO_4_ is present, leads to the worst analytical calibration figures. On the contrary, method C, where the insoluble BiPO_4_ phase is formed first on the carbon surface, and then covered by the PSS polymeric layer, shows the highest sensitivity; however, a high intercept value is observed, in particular with Cd, which is an indication of significant non-linearities at low concentrations. For the Bi/PO_4_/PSS (A) and Bi/PO_4_/PSS (B) electrodes, an intermediate behavior is observed; Method B is then characterized with a slightly better sensitivity than Method A, although, as it was discussed for the data reported in Table 2, a difference below 10% may be also ascribed to inter-electrode repeatability. In both cases, the non-linearity of the bismuth film at low concentrations of analyte is significantly reduced.

The explanation of such a behavior is related the preparation method, which tends to modulate the competition between the two equilibria, of (i) the complexation of Bi^3+^ and/or BiO^+^ ions with the –SO_3_^−^ Na^+^ ligands of the PSS structure; and (ii) the solubility product of BiPO_4_, which takes place upon drop-casting and drying of the deposition solution. The intermediate situation seen in Methods (A) and (B), where both equilibria are competing, appears to be the most suitable to form an active bismuth layer on the carbon surface, since the best properties of the two cases are synergically combined.

**Figure 4:**
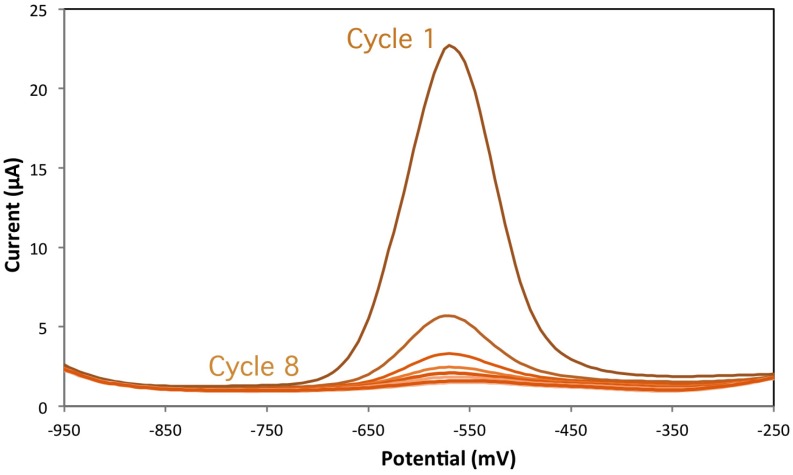
Decreasing intensity of the vanadium signal at −580 mV during the eight cycles in the activation step of Bi/VO_4_/PSS(B).

The effect of the coprecipitating anion was then investigated using the vanadate ion in place of orthophosphate. Insoluble BiVO_4_ is a well-known pigment with a bright yellow color, which is formed upon mixing sodium metavanadate and bismuth nitrate. Moreover, the electroactive behavior of vanadium may be exploited to give useful indication on the redox processes. Accordingly, a yellow color is observed upon casting and drying, as an indication that BiVO_4_ is formed on the surface of the screen printed electrode. In Figure 4, the DPV signals obtained during cycling the freshly-prepared Bi-VO4-PSS electrode under the activation phase are shown.

According to the well-known electrochemistry of vanadate ion [30], the signal around −580 mV is due to the redox behavior of vanadium (V) species. The signal is very intense during the first cycle, and then decreases rapidly until going off to zero after eight cycles.

This behavior indicates that the BiVO_4_ particles are reduced during the activation step to zerovalent bismuth and vanadium:

BiVO_4_ + 6 e^−^ + 8 H^+^ → Bi^0^ + V^0^ + 8 H_2_O

The zerovalent vanadium is reoxidized to vanadate during the anodic step, and then migrates into the bulk of the solution. In fact, vanadium is not known to preconcentrate on the surface of mercury or bismuth electrodes under stripping conditions. The previous suggestion on the slow kinetics of bismuth reduction from a solid phase is confirmed. Moreover, it appears that the cyclic activation process we have proposed with the stripping reduction step at −1.10 V, which does not require hydrogen “in statu nascendi” for bismuth reduction, is fully efficient to result in an active bismuth surface. The electrochemical experiment cannot, instead, provide any information on the fate of phosphate ions during reduction of BiPO_4_ particles. An investigation of the surface of the screen printed working electrode has recently been started using laser-ablation, inductively-coupled-plasma mass spectrometry (LA-ICP-MS). Preliminary results seem to indicate that no ^31^P is left on the surface after the activation step; this is in full agreement with the reduction of BiPO_4_ to Bi^0^ and PO_4_^3−^ which are removed from the negatively-charged surface of polystyrene sulfonate.

**Figure 5:**
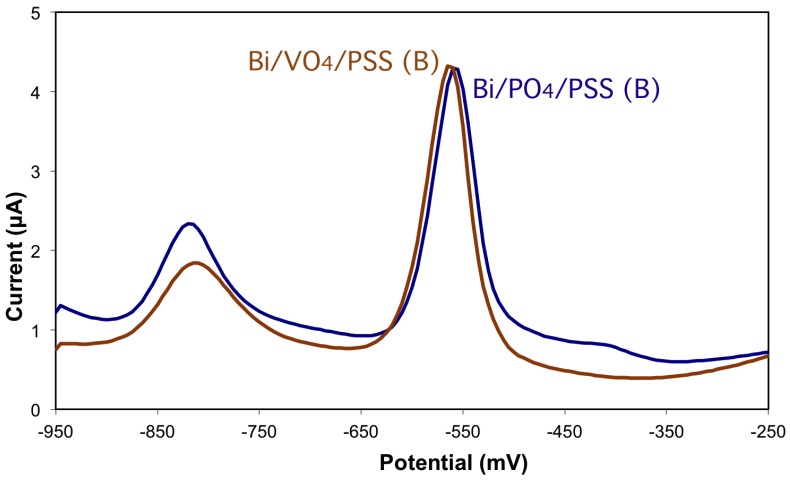
Comparison of differential-pulse stripping profiles of 50 ug/L Cd and Pb using Bi/PO_4_/PSS(B) and Bi/VO_4_/PSS(B) electrodes.

The electrodes prepared from BiPO_4_ (Bi/PO_4_/PSS(B)) and BiVO_4_ (Bi/VO_4_/PSS(B)) are then compared for the analysis of a 50 ug/L solution of Cd and Pb (Figure 5).

The electroanalytical behavior is very similar, showing the two peaks of similar shape and position in the potential scale, which are attributed to cadmium around −830 mV and lead around −560 mV. The analytical figures are also very similar, with the vanadate-based electrodes showing a slight decrease in sensitivity for Cd, and intercepts near zero, indicating a better linearity at low-concentrations.

Following the suggestion of Brainina [18] about the effect of bismuth loading on the analytical performance, two electrodes have been prepared using Method B with a 5-fold increase of bismuth loading, Bi/PO_4_/PSS(B5) and Bi/VO_4_/PSS(B5), and tested under the same experimental conditions. The Bi/PO4/PSS electrode at different bismuth loadings shows a similar electrochemical behavior; instead, the high-loading, VO_4_-based electrode looked quite different, with a significant shift to lower potential, and an evident shoulder around −600 mV. This feature is undoubtedly to be ascribed to vanadium, since in the activation step the vanadium feature at −580 mV is not reduced to zero even after 20 cycles. This observation is in full agreement with the proposal of a reduced diffusivity of metal ions inside the bismuth film. If the low-concentration Bi/PO_4_/PSS(B) and the high concentration Bi/PO_4_/PSS(B5) electrodes are compared for cadmium analysis, sensitivity is increased with bismuth loading, but at the expense of a significant non-linearity to low concentrations.

**Figure 6:**
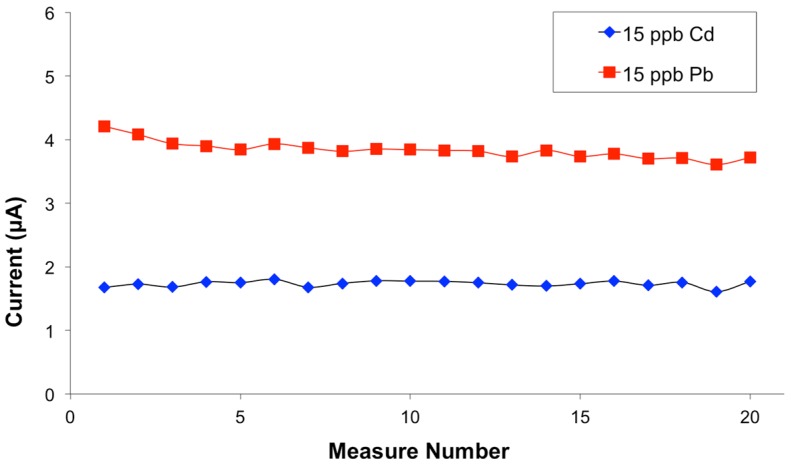
Long-term stability of cadmium and lead signals in differential-pulse stripping on a Bi/PO_4_/PSS(B) electrode.

Finally, the limits of detections are in the low-ppb range, with values between 0.6 and 1.1 μg/L for Pb, and between 1.1 and 1.6 μg/L for Cd. Electrode stability under analytical conditions has been evaluated for the low-concentration Bi/PO_4_/PSS(B) electrodes which, overall, show the best analytical figures for cadmium and lead. They are also characterized by good stability; a long-term study of a Bi/PO_4_/PSS(B) screen printed electrode is reported in Figure 6.

No decrease of the analytical signal is shown even after 20 cycles and no significant effect of exposure to air is observed. As a practical consideration, these electrodes, after deposition of the BiPO_4_ nanoparticles, are indefinitely stable in air, and they just require a simple activation in situ prior to the analysis.

## 4. Conclusions

The very rich coordination chemistry of bismuth aquo-ions [21] can be exploited to optimize the analytical performance of bismuth film screen-printed electrodes. An oxidative pretreatment of the surface of the working carbon electrode was confirmed to increase the reduction kinetics of Bi^3+^ to Bi^0^ via the coordination and grafting of Bi^3+^ and/or BiO^+^ species to –COO^−^ groups of the conductive carbon surface, and the creation of a preferential route for electron exchange. The use of basic sodium carbonate solutions proved very effective in optimizing the reactivity of the bismuth film, with a substantial increase in the analytical sensitivity, at the expense of a strong reactivity with atmospheric oxygen, leading to Bi_2_O_3_ particles which cannot reform the active bismuth surface.

Such chemical interactions are also taking place with the active sites of the surface of the polymeric film; in this way, the polymeric layer plays an active role, and the mechanical stability of bare screen-printed electrodes can be profitably used. The sodium salt of polystyrene sulfonate polymer (PSS) appeared to be particularly suited to form and stabilize an active bismuth film thanks to its –SO_3_^−^ Na^+^ groups which may mimic the surface sites formed upon electrochemical oxidation in sodium carbonate. We are convinced that applicability of these bismuth-surface interactions may be useful for other electrodic materials, primarily glassy carbon, where their more uniform surface structure and the absence of any residual organic contaminant from inks may lead to new analytical perspectives.

Moreover, PSS was found to have a beneficial effect even using the Brainina’s route to form a nanophase of an insoluble bismuth salt as precursor of the bismuth film. In this case, reduction occurs at the surface of the BiXO_4_ (X = P,V) particles via removal of the anion, as was evidenced by the complete loss of the vanadium signal during the activation step. The increase of the analytical sensibility for trace metals is, however, accompanied by non-linearity at low concentrations of analytes, which become more evident by increase in the bismuth loading on the electrode surface.

The best analytical performance is then gained by a careful choice of the preparation route, since the competition between the two equilibria, (i) of grafting Bi^3+^ ions onto the R-COO^−^ ligands of the PSS structure, and (ii) the solubility product of BiXO_4_ (X = P,V), is leading to a synergic combination of a BiXO_4_-derived metallic film, which is still interacting with the active sites of the polymeric layer. In particular, the electrodes prepared via method B (Bi/PO_4_/PSS(B)) show good analytical figures for the analysis of cadmium and lead, either in terms of sensitivity and of limits of detections, and a good stability under analytical conditions. The use of BiVO_4_ as bismuth precursor led to similar results, but the need of carefully removing all vanadate from the electrode surface tends to favor the use of phosphate-based electrodes. Work is in progress to test PSS-based electrodes under real conditions of analytical samples to compare their performance with Nafion-based SPEs.

## Figures and Tables

**Table 1:** Comparison of the calibration linear best fit parameters for Cadmium analysis as a function of the chemical pretreatment of the screen printed electrode. Experimental conditions: [Cd^2+^] additions = 12 μg/L; 60-s stripping; differential pulse voltammetry.

Pretreatment of the Graphite Working Electrode	Best Fit Using All Calibration Points		Best Fit Using Calibration Using Calibration Points above 12 μg/L	
	Slope	Intercept	Slope	Intercept
No pretreatment (BiFE)	1.008	−4.698	0.996	0.421
Treatment A (Type-A BiFE)	3.130	−5.838	2.947	−0.122
Treatment B (Type-B BiFE)	5.893	−7.02	5.738	−0.174

**Table 2:** Comparison of the calibration linear best fit parameters for cadmium and lead analysis as a function of the polymer used for casting. Experimental conditions: [Cd^2+^] additions = 25 μg/L; [Pb^2+^] additions = 25 μg/L; 60-s stripping; differential pulse voltammetry.

Bi-Polymer Film	Cadmium			Lead		
	Slope	Intercept	LOD	Slope	Intercept	LOD
Bi/Naf	1.098	−1.698	1.2	0.984	−0.618	0.8
Bi-Meth	N/A	N/A	N/A	0.346	−1.80	N/A
Bi-PSS	2.911	−8.172	1.1	4.262	−0.972	0.9

**Table 3:** Comparison of the linear calibration best fit parameters for cadmium and lead analysis as a function of the method used for bismuth film preparation. Experimental conditions: pH = 2; [Cd^2+^] additions = 25 μg/L; [Pb^2+^] additions = 25 μg/L; 60-s stripping; differential pulse voltammetry.

Electrode Type	Cadmium			Lead		
	Slope	Intercept	LOD	Slope	Intercept	LOD
Bi/PO_4_/Naf	3.49	1.53	1.6	2.72	−4.19	1.1
Bi/PO_4_/PSS(A)	4.44	−10.47	1.2	8.46	−6.29	0.8
Bi/PO_4_/PSS(B)	4.998	−9.49	1.1	12.31	−10.82	0.6
Bi/PO_4_/PSS(C)	6.031	−23.01	1.0	8.64	−17.09	0.8
